# Levan-Capped Silver Nanoparticles for Bactericidal Formulations: Release and Activity Modelling

**DOI:** 10.3390/ijms20061502

**Published:** 2019-03-26

**Authors:** Álvaro González-Garcinuño, Rubén Masa, María Hernández, Ángel Domínguez, Antonio Tabernero, Eva Martín del Valle

**Affiliations:** 1 Department of Chemical Engineering, University of Salamanca, 37008 Salamanca, Spain; alvaro_gonzalez@usal.es (Á.G.-G.); ruben.masa.gonzalez@gmail.com (R.M.); mariahg93@hotmail.com (M.H.); antaber@usal.es (A.T.); 2 Department of Microbiology and Genetics, University of Salamanca, 37008 Salamanca, Spain; ado@usal.es; 3 Institute for Biomedical Research of Salamanca (IBSAL), University of Salamanca, 37007 Salamanca, Spain

**Keywords:** silver, nanoparticles, levan, modelling

## Abstract

An environmentally friendly technique was used to produce levan-capped silver nanoparticles of about 30 nm (with a loading of 30%) that showed bactericide effect, for *E. coli* and *B. subtilis*. That effect was mathematically studied with a dose-response model (lethal dose of 12.4 ppm and 6.8 ppm respectively). These silver nanoparticles were subsequently introduced in a gel to create a silver release system with bacteria inhibition activity. Silver release from the gel and its bactericidal activity was theoretically studied to develop a unique model that is able to predict accurately both silver release and lethal dose for any type of bacteria. This model will be useful for performing predictions for future silver in gel applications.

## 1. Introduction

Silver nanoparticles antibacterial properties and the problems concerning its proper release have already been known for decades. Due to this fact, silver controlled release systems, such as liposomes or hydrogels, can be considered as potential formulations for biomedical uses, such as wound dressing [1].

Several techniques have been used for obtaining silver nanoparticles, such as metal salt reduction with organic compounds or with polyoxometalates [2,3,4]. Nevertheless, the use of organic compounds can be environmentally toxic as well as providing biological risks and as a consequence, green techniques have been developed to overcome these limitations. One of the main processes involves the use of biopolymers and/or polysaccharides. As an example, starch [5], chitosan [6], or sucrose can be useful as reducing agents [7].

Among this type of polymers, levan is a fructose homopolysaccharide with proper properties for biomedical applications [8,9,10,11,12]. Levan is obtained by cultivating a bacterial strain (*Zymomonas mobilis* or *Bacillus subtilis* for instance) under acidic conditions (pH between 5–6) and high sucrose concentrations. Under these experimental conditions, the microbial strain releases an enzyme, levansucrase, which breaks the bond glucose-fructose and polymerizes subsequently the fructose to obtain the polymer [8].

Levan has been able to act as a reducing and capping agent for gold and silver salt solutions to obtain the respective polymer-capped nanoparticles [13]. However, to the best of our knowledge, the only work that used levan has been focused on the nanoparticles effect as catalyst and did not study the bactericidal effect of the silver nanoparticles, without a quantification of the amount of silver included in the levan nanoparticles [13]. That fact is important concerning biomedical applications because silver can be toxic for the human body and its exact amount in the pharmaceutical formulation, nanoparticles in this case, must be known. Previous article also indicated metal nanoparticles size with TEM (Transmission electron microscopy) images but particle size distribution was not deeply studied.

Silver, as it was mentioned before, must be included in a drug release system to be used in a proper way. Silver nanoparticles have been included in liposomes (about 20 nm) [14], in phospholipids to enhance nanoparticles cytotoxicity against cancer cells [15] or in an antimicrobial glass [16,17]. Also it is possible to include silver nanoparticles in a gel formulation, which shows great potential for wound dressing applications [18]. Specifically, alginate is one of the main polymers that have been used for that purpose because the polymer can promote tissue formation and healing [19].

Some authors have proposed the inclusion of silver nanoparticles into an alginate gel. Rescignano et al. [18] prepared a nanocomposite hydrogel alginate-silver and they evaluated their rheological and bactericidal properties with two Gram negative bacteria (*Escherichia coli* and *Pseudomonas aeruginosa*). Their results indicated that this system can be useful for inhibiting bacteria growth as well as highlighting the experimental conditions to obtain the highest elastic modulus. Obradovic et al. [20], incorporated silver into discs of a polymeric mixture alginate/PVA. They studied the mechanical properties of the systems and the bactericidal effect with *E. coli*, obtaining positive results.

However, the whole drug delivery system, combining silver release and silver bactericidal effect, has not been studied yet. It is possible to find in literature some papers concerning the mathematical analysis of the silver dose for achieving a bacteriostatic effect [21], or focused on silver release modelling from alginate hydrogels [22]. Those works did not study both phenomena simultaneously and thus cannot provide a global model for predicting the effect of silver nanoparticles on bacteria after its release from an alginate hydrogel.

Based on the previous considerations, the main objectives of our work are the following: The first one is to prepare and to characterize levan-capped nanoparticles with its bactericidal evaluation for both Gram positive and Gram negative bacteria. The second one is to include these nanoparticles in an alginate gel to create a drug delivery system that will be studied mathematically by combining silver release and silver dose for bactericidal effect. The developed mathematical model will be helpful to reproduce phenomena that can be important regarding silver-in-gel further applications, such as wound dressing.

## 2. Results

### 2.1. Characterization of Nanoparticles Obtained

**Figure 1:**
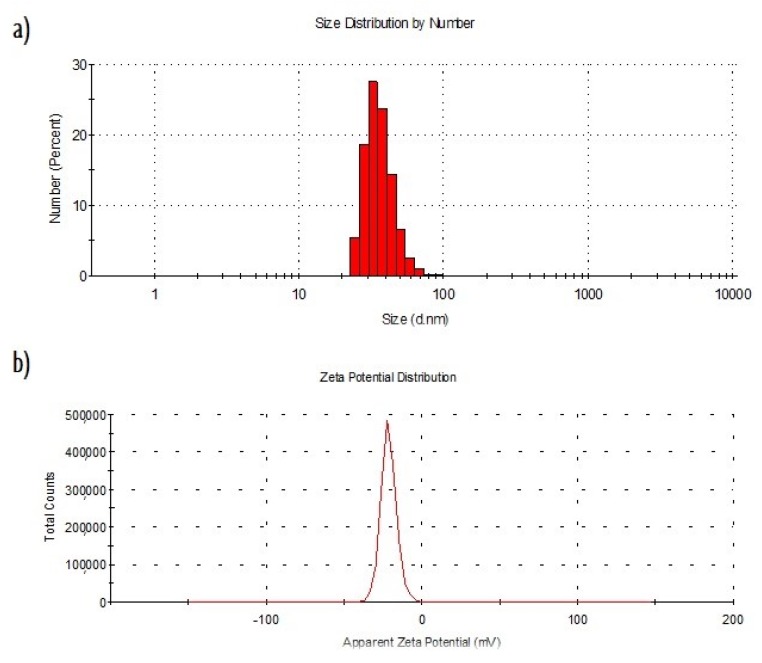
Characterization of the AgLeNPs. (**a**) particle size distribution determined by DLS. (**b**) Zeta potential distribution determined by DLS.

By following the protocol described in Section 3.1, AgLeNPs were obtained and characterized by Dynamic Light Scattering (DLS), TEM, UV-visible and Fourier-Transform Infrared Spectroscopy (FT-IR). Results are illustrated for an initial silver concentration of 125 ppm. Figure 1 illustrates the particle size distribution (a) and the Zeta potential distribution (b) of the levan-capped silver nanoparticles.

**Figure 2:**
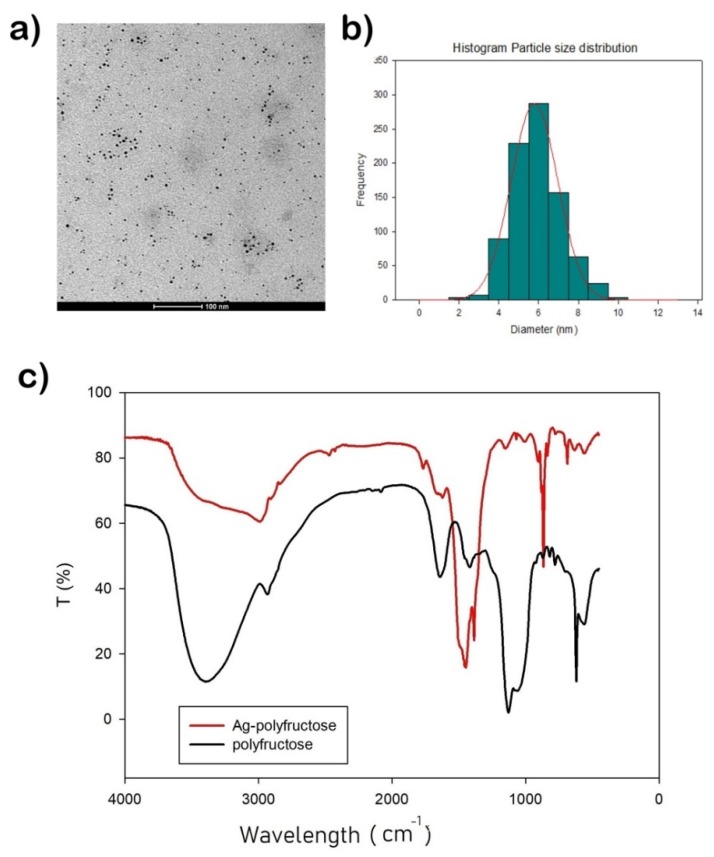
(**a**) Transmission electron microscopy (TEM) image of the nanoparticles synthetized. (**b**) Histogram of particle size distribution measured by Image J. (**c**) Fourier transform infrared (FT-IR_ spectra for AgLeNPs (red) and its comparison with only polyfructose chains (black).

An average nanoparticle size of 36.9 ± 11.8 nm was obtained. Besides, nanoparticles show a significant negative surface charge (−22.3 ± 5.8 mV) which prevents particle aggregation phenomena due to repulsive forces and therefore, it suggests a proper nanoparticles stability. Particles have negative charge because silver ions lose their charge because of the reduction reaction with polymer. This value is in accordance with others presented before (range from −20 to −25 mV) [23,24]. Figure 2a shows TEM image of AgLeNPs where their sphericity and their monodispersion are observed. TEM image shows particles of about 6 nm, that is a smaller size in comparison with the Particle Size Distribution (PSD) from dynamic light scattering (results from ImageJ analysis are given in Figure 2b). That size reduction is attributed to the dehydration process for microscope size preparation, as was indicated in Reference [25]. Another phenomenon to explain this size reduction is that TEM cannot detect the polymer layers but can indicate the silver cores. Our nanoparticles size and shape are similar to the obtained nanoparticles in Reference [13]. Figure 2c shows the infrared spectrum of the levan (black) and the levan-capped silver nanoparticles (red). Levan spectrum matches with the typical spectrum for this polymer [26]. When silver is added, fingerprint peaks (1200–900 cm^−1^) that correspond to stretching vibration of C–OH and C–O–C bonds are modified. That fact can indicate a possible interaction polymer-silver.

**Figure 3:**
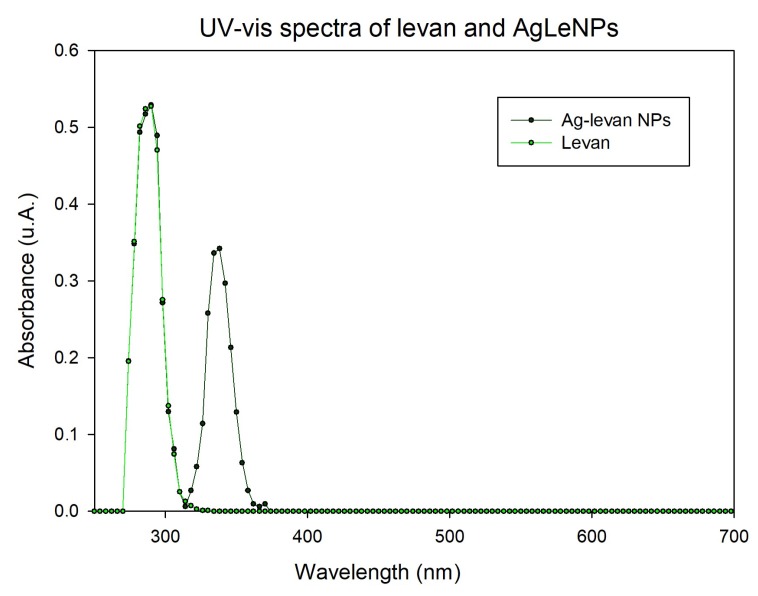
UV-vis spectra of levan and AgLeNPs.

UV-visible experiment (Figure 3) shows a peak in common (silver nanoparticles and polymer alone) around 290 nm and another peak (less intense), around 350 nm. That peak is slightly lower than the surface plasmon resonance at 400 nm because nanoparticles size and the refraction index (medium with NaOH and levan) of the system can modify the position of the surface plasmon resonance peak [27]. On the other hand, it is possible to observe that there is no absorption peak for the polymer alone at that wavelength. This figure shows that these nanoparticles are formed by a polymer-silver.

The amount of silver into the nanoparticles was estimated by measuring the supernatant by ICP-MS, obtaining as a result a concentration of about 7.1 ± 2 ppm. Considering the initial concentration of silver in the reaction (125 ppm), the entrapment yield was 94.3 ± 1.6%. After that, nanoparticles were washed by using distilled water and centrifuged again to remove the silver ions non-specifically adsorbed. The supernatant was measured again by ICP-MS and the concentration was 1.3 ppm which is in the range of the standard deviations. Therefore, the non-specific adsorption of silver ions could be considered as negligible. Loading was calculated as the mass of silver per mass of levan. Considering the concentration of levan (400 ppm), the loading capacity takes a value of 29% (mass silver/mass levan).

### 2.2. Bacteria Survival

**Figure 4:**
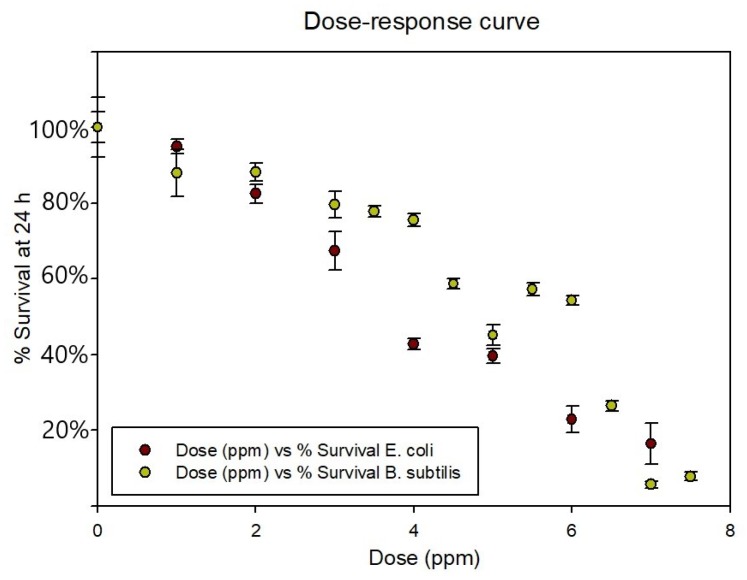
Dose- response curve for *E. coli* and *B. subtilis* with AgLeNps.

Firstly, it was determined the effect of AgLeNPs, previously to be included in the gel formulation and the results were used to determine the parameters n and D_0_ in the model proposed (Equation (10), single hit-multiple target model). Figure 4 represents the percentage of survival (related to control) at different doses of silver in nanoparticles by measuring the increase in absorbance after 24 h’ culture. It is important to specify that the silver dose in the figure (x-axis) corresponds to the amount of silver in the solution that was added inside the AgLeNPs.

The survival curves from Figure 4 suggest more effectiveness in stopping bacterial growth in gram-negative than gram-positive bacteria. This difference agrees with the results from [28,29]. The effect on Gram negative bacteria is more intense because it can be associated with the penetration of the colloid into the cytoplasm, with the subsequent local interaction of silver with cell components causing damages to the cells. However, on Gram positive bacteria the thick peptidoglycan layer of the cell wall prevents the penetration of the NPs inside the cytoplasm and therefore, the antimicrobial effect is limited and seems related to the interaction with the bacterial surface. The dose used for inhibit bacteria growth (Minimum inhibitory concentration) is smaller than other (13 ppm) doses previously reported for *E. coli* [30]. Significant differences between the results for the different bacteria were highlighted after performing the Wilcoxon Test (See Supplementary Material).

From Figure 4 and with equation 1 linearization, it is possible to calculate the parameters related with bacterial survival. The fitting results show similar values for the number of targets (n): 1.904 for *B. subtilis* and 1.868 for *E. coli*. However, the differences in action mechanism are explained by the different lethal doses (D_0_) calculated: 12.6 ppm for *B. subtilis* and 6.8 ppm for *E. coli*. (See Supplementary Material for survival curves fitting).

**Figure 5:**
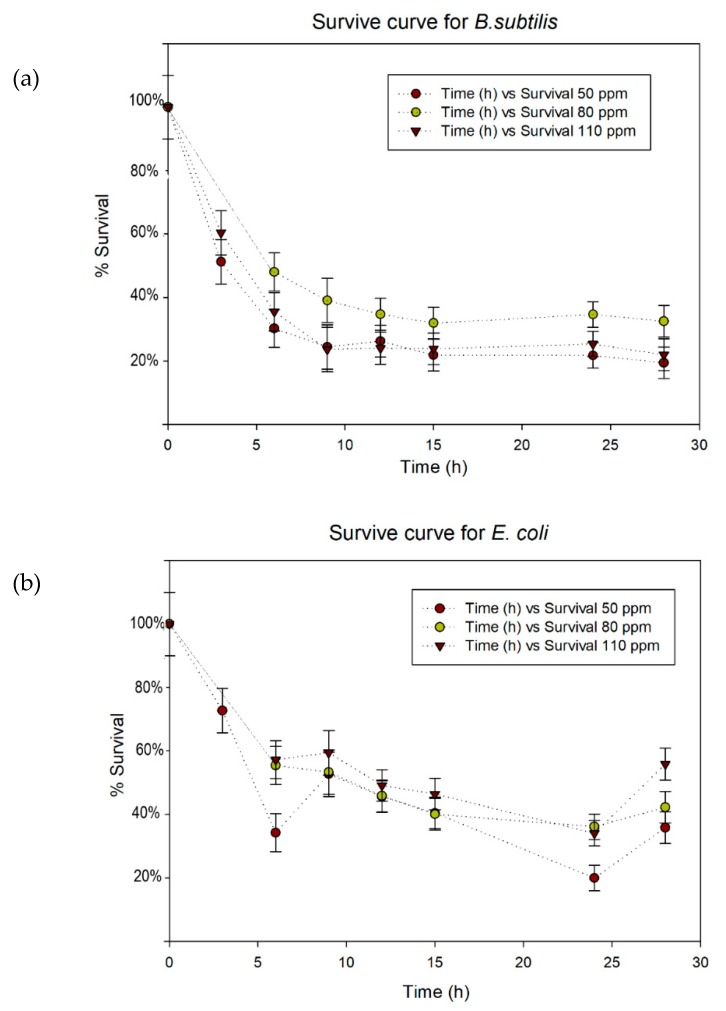
Survival curves for *B. subtilis* (**a**) and *E. coli* (**b**).

Secondly, it was studied the system with silver nanoparticles embedded in an alginate gel. Three different concentrations of silver in the gel (50, 80, 110 ppm) were assayed and the evolution of bacterial survival at different times is showed in Figure 5, where the first graph represents the evolution for *B. subtilis* and the second graph the evolution for *E. coli*.

In order to analyse if silver is released as nanoparticles or as ions, DLS measurements were performed to the medium culture previous to adding the gel and after 20 hours of culture. The results obtained showed the same particle distribution size (around 35 nm) as the one reported in Figure 1a and an increase in the intensity (proportional to the nanoparticle concentration) from 61.5 ± 4.1 kcps to 139.6 ± 5.8 kcps. This increase highlights the existence of light scattering that is explained by the release of nanoparticles from the gel.

The statistical analysis of Survivals curves (Wilcoxon test) showed that there were not significant differences between the concentrations examined (see Supplementary material). That fact indicates that there are not differences in AgNPs concentration in the well. Therefore, the process is only controlled by the release from the gel and doses around 5 ppm (dilution 1:10 of gel concentration 50 ppm) are enough to stop the bacterial growth in both strains, with a survival percentage below 40%.

### 2.3. Parameters Estimated

Parameters k and n were estimated for the experiments in Figure 5a,b. Therefore, 6 independent experiments, with two different strains were used to fit the model to the experimental data. Table 1 shows the value of these two parameters for each experiment, the time for estimation, the number of iterations needed, the weighted residuals and the chi-square value. The parameters k and n (combined) represents the rate for release. K is a constant that involves the structural characteristics of the gel, whereas n is the indicator of release mechanisms. If *p* = 1, the release follows a zero-order kinetics; if *p* < 0.5 (our study-case), the release is controlled by Fickian diffusion [22].

Results of parameter estimation showed that in all cases weighted residuals are smaller than chi-square values, which indicates a good fit for the experimental data. The number of iterations and the time for parameter estimation indicates that the model is well-posed. From parameters estimated, *p* is assumed to have a value from 0.11 to 0.26 for all systems. For parameter k, four estimations showed values around 0.2 whereas two of them calculated higher values (around 0.8). Despite these differences, model can fit for all experimental data if an average value of k and p is applied (0.206 for k and 0.193 for n). The average standard error was around 9%.

**Figure 6:**
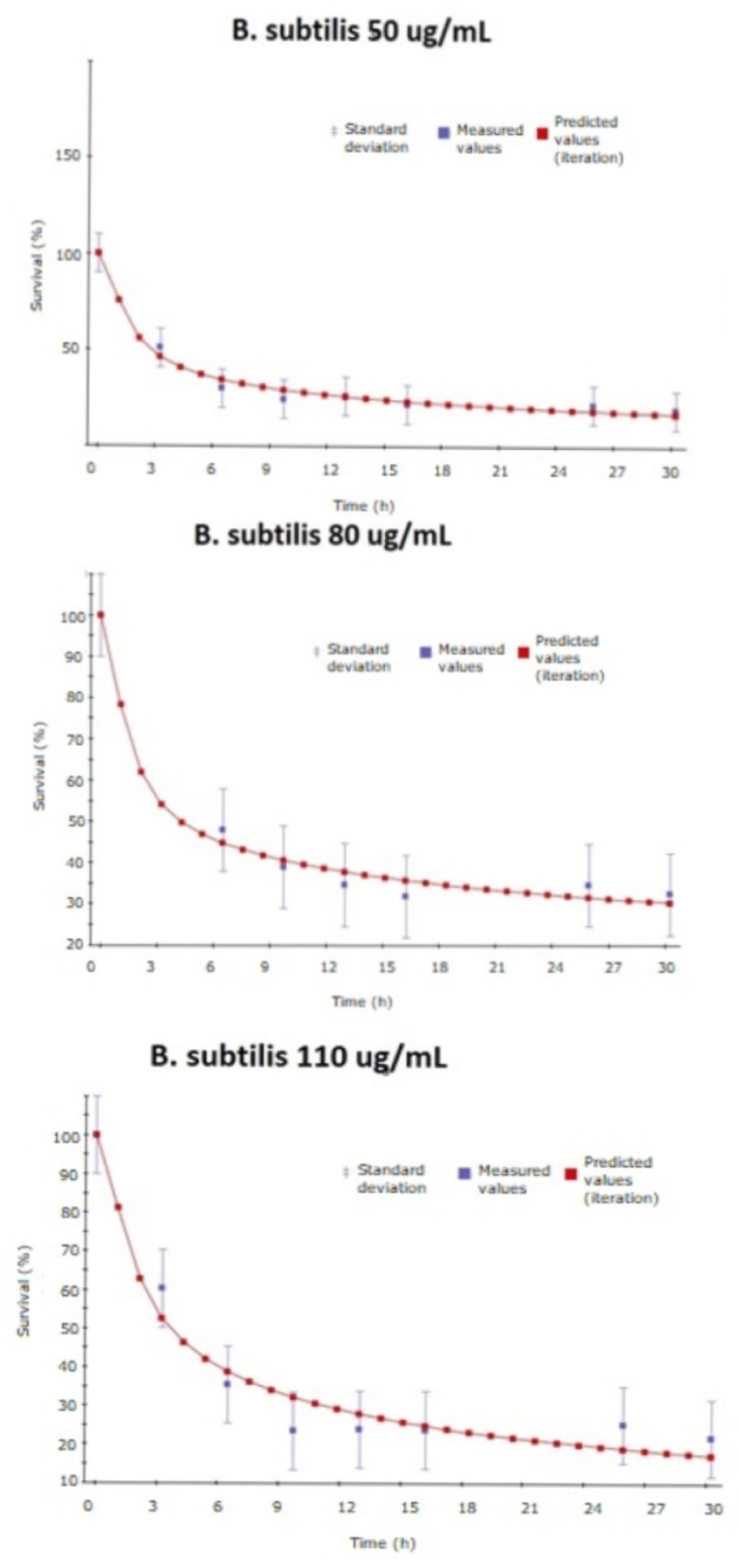
Experimental data and model prediction fitting for *B. subtilis*.

**Figure 7:**
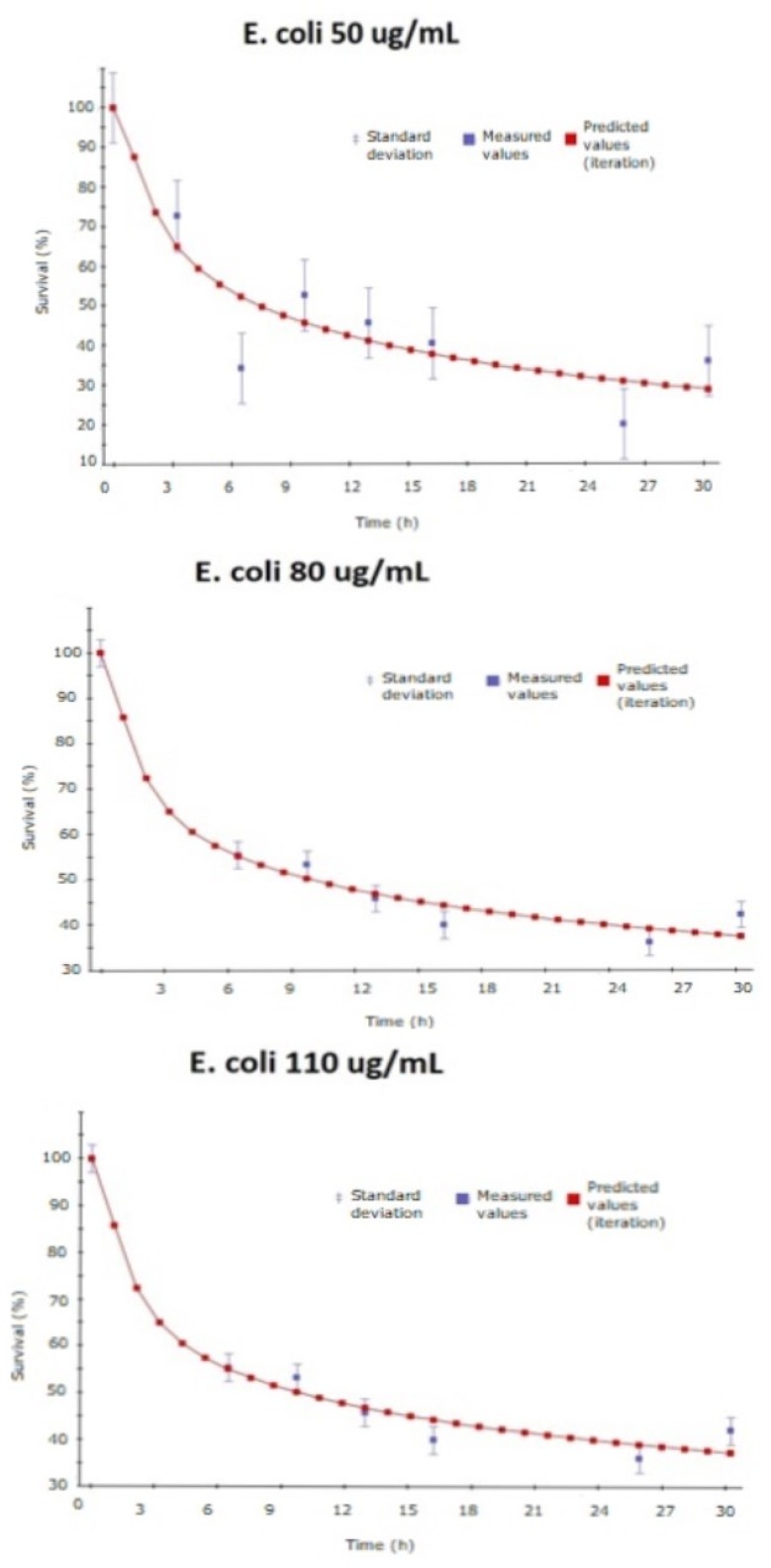
Experimental data and model prediction fitting for *E. coli*.

Figure 6 represents the fitting (performed by gPROMS) for the experiments of survival in *B. subtilis* and Figure 7 in *E. coli*. In both cases, the experimental data and the variance set by the simulation are represented in blue and the fitting curve predicted is represented in red.

### 2.4. Simulations from Model

With the average value for k and n, more simulations were carried out in gPROMS in order to know the behaviour of other variables considered in the model. Table 2 represents the values of the parameters used for simulations and its reference or calculation.

**Figure 8:**
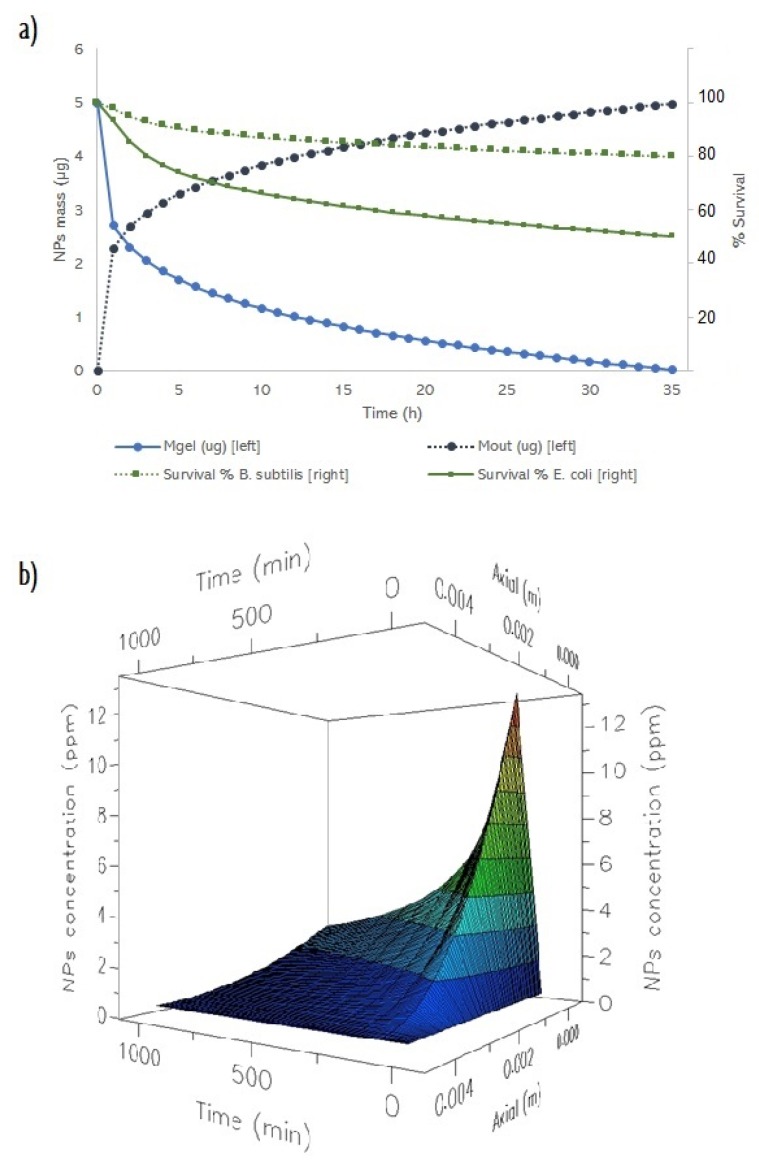
(**a**) Profiles obtained by simulations from the model. Evolution of nanoparticle (NPs) mass inside the gel and outside and the evolution of survival percentage for 35 hours in *B. subtilis* and *E. coli*. (**b**) evolution of nanoparticles concentration inside gel depending on time and position.

Finally, Figure 8 shows the performed simulations for a concentration of 50 µg/mL in the gel, using *B. subtilis* and *E. coli*. In the right axis is represented the mass of particles released (blue circles–straight line) and the mass of particles remained (blue circles–dashed line) in the gel and left axis represents the survival percentage for each strain (green square–straight line for *B. subtilis* and green squares–dashed line for *E. coli*). The model is able to predict accurately the release profile. However, significant differences where obtained for the survival rate of *B. subtilis*. In spite of that fact, the model can predict survival tendency, given as a result a higher *B. subtilis* survival than the *E. coli* survival. This phenomenon agrees with the experimental data. In this context, it is important to specify that these simulations (Section 2.4) were carried out with an average value for k and p (obtained in Section 2.3) in order to get a global model for any strain. That is the reason why survival prediction was not accurate for *B. subtilis*. Values for k and p should be used from Table 1 for every condition if a more precise simulation must be done.

Finally, Figure 8b represents the evolution of NPs concentration (z-axis) along the gel (y-axis) at different times (x-axis). This 3D graph represents how diffusion controls the movement of nanoparticles inside the gel and how release is controlled by this process, creating a gradient of concentration (axial dispersion). These simulations can be used in further studies in order to optimize the release of silver nanoparticles from a gel for topical administration.

## 3. Materials and Methods

### 3.1. Nanoparticle Synthesis and Characterization

Levan was synthesized in a cell-free system by using the enzyme Fructosyl-transferase from *Bacillus subtilis* (S168), purchased from Creative Enzymes. The reaction was carried out in a batch stirred tank reactor (volume 100 mL) at 150 rpm and 37 °C (thermostatically controlled) for 24 h by using 90 g/L sucrose as substrate (Sigma Aldrich) and 0.2 ppm enzyme (as catalyst). In order to precipitate the polymer, ethanol 96% (VWR Chemicals) was added to reaction medium in a proportion 3:1 (volume:volume) and calcium chloride (Sigma Aldrich, St. Louis, MO, USA up to a concentration 1 mM. The mixture was kept at −20 °C for 24 h to provoke the polymer precipitation. After that, supernatant was discarded by centrifugation at 10.000 rpm for 10 min. The precipitate was dried by lyophilization at 0.050 mbar and −55 °C (Telstar, Spain).

Levan-capped silver nanoparticles were produced by dissolving 20 mg of the obtained levan in 49 mL of basic water solution (NaOH 0.2% *w*/*w*). Then, 1 mL of a solution AgNO_3_ (1 mM), previously prepared, was added to the polymeric solution. The new mixture was stirred at 200 rpm during 10 min at 25 °C for silver nanoparticles formation. This methodology was used in Reference [13]. Nanoparticles were subsequently separated from the non-reactant medium by centrifugation at 10,000 rpm for 10 min. Finally, levan-silver nanoparticles (AgLeNP) in the pellet were dried by lyophilization (0.050 mbar and −55 °C) for further characterization experiments.

In order to characterize the polymer capped silver nanoparticles, Fourier-transform infrared spectrum were carried out by using a Perkin-Elmer Spectra One Instrument. Potassium bromide (KBr) pellets were used and 32 spectra were recorded with a nominal resolution of 4 cm^−1^.

Surface-plasmon-resonance effect was determined by using a UV visible equipment, (UV-1800, Shimadzu).

Nanoparticles were resuspended in phosphate buffer saline (PBS) for particle size distribution and surface-charge (Zeta potential) measurements. Both analyses were performed by Dynamic Light Scattering using Malvern ZS Nano as equipment.

Nanoparticles shape and morphology were studied by Transmission Electron Microscopy (TEM) in a Zeiss EM902 at 80 kV after loading a droplet of the suspension on a grid with a subsequent drying process with air.

TEM image analysis was performed with ImageJ. A threshold modelling (white/black) was carried out previous the particle counting and then results were processed with Mathematica to avoid noise problems.

### 3.2. Silver Determination in the Polymer-Capped Nanoparticles

The amount of silver in the nanoparticles was determined by difference after analysing the mass of silver in the supernatant after the centrifugation step. Silver was quantified by Inductively Coupled Plasma Mass Spectrometry (ICP-MS) using an instrument model 7800 (Agilent Technologies, Santa Clara, CA, USA). The mass used for silver was 107 with Radiofrequency Power 1500 Watts and the pump operating at 0.10 rps. The flow rates for gases are the following: 15.0 L/min (plasma gas), 0.90 L/min (auxiliary gas), 0.99 L/min (nebulization gas). For equipment calibration, different concentrations (from 0.2 to 10 ppm) were prepared from a Certificate standard solution of silver (concentration: 1000 mg/L).

### 3.3. Gel Formation

Silver-polymer nanoparticles were included in an alginate solution (2% *w*/*w*) in order to study the behaviour of particles inside a drug delivery system formulation. Different concentrations of nanoparticles were included in the gels in order to study the effect of the concentration in its release and its bacteriostatic effect. Alginate solution containing AgLeNPs was reticulated by adding 60 µL calcium chloride (2% *w*/*w*) per each millilitre of alginate solution. The gelation process is instantaneous [31].

### 3.4. Bacteria Survival Assays

Two strains were selected to study the bacteriostatic effect of the AgLeNPs obtained. *Bacillus subtilis* (ATCC 6051) was selected as example of Gram-positive bacteria and *Escherichia coli* (strain K12) as Gram-negative. Both bacteria were cultured in Agar-Nutritive plates to obtained isolated colonies at 30 °C. Then, one colony of each bacterium was suspended in liquid medium agar-nutritive to proliferate for 6 hours before performing the survival assay. After these six hours, optical density (O.D.) was measured by spectrophotometry at 600 nm and the appropriate dilution was carried out in order to get the bacteria for seeding at 0.1 O.D.

Firstly, survival assays were performed with free AgLeNPs in the medium. This assay was carried out in 24-microwell plates, where the proportion of solutions added to each well (1 mL final volume) is the following: 100 µL solution of AgLeNPs at different concentrations, 100 µL solution of bacteria at 0.1 O.D., 800 µL culture medium. Two different controls were used: one control with bacteria without nanoparticles and another control with culture medium without bacteria. Each concentration was studied by triplicate. Absorbance at 600 nm was measured at time 0 and 24 h by using Microplate Reader: EZ Read 2000 (Biochrom, Cambridge, UK) and the survival percentage for each concentration was calculated by using the control value (without nanoparticles and a survival value of 100%) as a reference.

Secondly, the experiments were carried out by adding 100 µL alginate solution containing AgLeNPs (at different concentrations) at the bottom of each well and the subsequent addition of calcium chloride to gel formation. Bacteria were added at 0.1 O.D. and culture medium up to final volume 1 mL in each well. Absorbance at 600 nm was measured at time 0, 3, 6, 9, 12, 15, 24 and 28 h for study the release from gel and its effect on both bacteria strains. Microwell plates were kept at 35 °C and 150 rpm in orbital shaker. Each concentration and time was studied by triplicate.

In both cases, Wilcoxon Signed Rank Test was used to determine if survival curves and dose-response curves are significantly different. Statistical method was carried out following the description showed by F. Wilcoxon [32]. According to Wilcoxon Test tables, critical value for seven trials (seven different times or doses measured) is 3, for a significance level of 0.05. Therefore, results will be significant between both curves (data serial) if the obtained value is smaller (or equal) than 3 (in absolute value).

Moreover, in order to check that silver is released as nanoparticles and not as ions, the intensity and the size of particles in the medium (previous and after including them inside the gel) was measured by DLS. With both values is possible to analyse in which form silver is released because light scattering intensity is directly proportional to nanoparticle concentration.

### 3.5. Model Formulation

**Figure 9:**
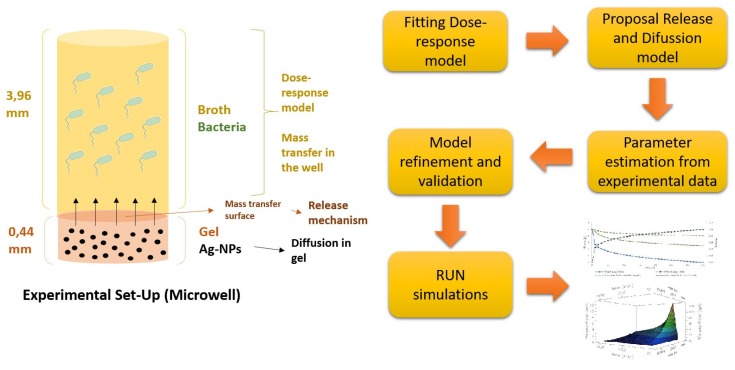
Experimental set-up and steps of modelling.

The model try to explain the behaviour of nanoparticles inside the gel, nanoparticles release from gel to liquid medium in the well and its toxicity effect on bacteria (survival reduction). The model was implemented in *gPROMS* 5.0.2 (PSE), containing 115 total equations (111 algebraic, 4 differential). For studying axial dispersion, gel was discretized on axial (z) in 100 beans. No discretization method was used for culture medium because mixing conditions guarantees no effects of concentration gradient in the liquid part of the system. The model takes 3 s to run in a personal computer Intel Core I3 3.70 GHz and 4 GB RAM memory. Figure 9 shows the dimension of the silver-gel and the steps to build the model, its validation and the parameter estimation of the release from the survival data.

#### 3.5.1. Nanoparticles Effect on Bacteria, Survival Modelling

Firstly, the effect of AgLeNPs on bacteria was modelled by using the results obtained with free dispersed particles in culture medium. Three different models for explaining the dose-response were studied: single-hit/single-target; multiple-hit/single-target; single-hit/multiple-target [33]. After fitting the experimental data to these models, best results were obtained with a single-hit/multi-target model, that explains the survival percentage depending on concentration or dose (Equation (1)). Fitting results and constant determination (D_0_ and n) can be seen in Supplementary Material.

(1) $$S=1-{\left(1-e^{-\frac{D}{D_{0}}}\right)}^{n}$$

The following assumptions were considered:

- Each cell has n targets
- Each target is inactivated by one nanoparticle
- The inactivation of 1 target is considered as “sub-lethal” event.
- All targets should be inactivated to kill the cell

In the heterogeneous system (gel and the liquid culture medium), the concentration in bacteria’s surface (C_s_) was considered as D (dose) for survival prediction because that dose will reach the gel surface and will be taken up by the microorganims.

#### 3.5.2. Mass Transfer of Nanoparticles in Gel

At the beginning of the experiment, after gel preparation, nanoparticles are distributed homogeneously. However, axial dispersion phenomenon occurs subsequently due to the nanoparticles release (the release surface is the upper part of the gel) to the liquid medium. The number of particles released is related to the number of particles available for release in the interface, which is controlled by Fick diffusion (Equations (2) and (3)):

(2) $$\frac{N_{\mathrm{NPs}}}{A}=-D_{\mathrm{ef}}\frac{{\mathrm{dC}}_{\mathrm{NPs}}}{\mathrm{dz}}$$

(3) $$\frac{{\mathrm{dN}}_{\mathrm{NPs}}}{\mathrm{dz}}+\rho_{\mathrm{gel}}\cdot \pi \cdot r_{w}^{2}\cdot v_{r}=0$$

where v_r_ is the velocity for the release and it is defined as the variation in concentration inside the gel with respect to time (Equation (4))

(4) $$v_{r}=-\frac{{\mathrm{dC}}_{\mathrm{in}}}{\mathrm{dt}}$$

By substituting Equation (1) in Equation (2), it is obtained the final expression that explains the concentration gradient through the gel (Equation (5)).

(5) $$\pi \cdot r_{w}^{2}\cdot \left(\rho_{\mathrm{gel}}\cdot v_{r}-D_{\mathrm{ef}}\cdot \frac{d^{2}C_{\mathrm{NPs}}}{\mathrm{dz}}\right)=0$$

The velocity of release can be estimated by following the Korsmeyer-Peppas model (Equation (6)) [34].

(6) $$\frac{{\mathrm{dM}}_{\mathrm{out}}}{\mathrm{dt}}=M_{\mathrm{tot}}\cdot k\cdot p\cdot t^{p-1}$$

where mass balance follows Equation (7),

(7) $$M_{\mathrm{in}}+M_{\mathrm{out}}=M_{\mathrm{tot}}$$

Parameters k and p from Equation (6) are responsible of the release velocity and they will be estimated by parameter estimation (Section 3.3). As was mentioned before, silver release was estimated by bacteria survival (see Section 3.3).

Cin can be calculated by knowing M_in_ and the gel volume (100 µL), Therefore, velocity of release is calculated with Equation (4).

#### 3.5.3. Mass transfer of Nanoparticles in Liquid Culture Medium

The behaviour of AgLeNPs in culture medium can be explained by using a correlation with dimensionless numbers, proposed by Doig et al. [35] for microwell plates (Equation (8)).

(8) Sh=0.19·Re0.68·Sc0.36

From Sherwood number (Sh), it is possible to determine the coefficient for mass transport in the liquid (Ks). Moreover, in order to perform an appropriate calculation of Sh and Sc number, the diffusion coefficient (Dif) for nanoparticles was calculated by using the Stokes-Einstein equation, taking the value from the DLS measurement as the hydrodynamic radius (Equation (9)).

(9) $$\mathrm{Dif}=\frac{k_{b}\cdot T}{6\pi \cdot µ\cdot r_{H}}$$

The time-dependent concentration of nanoparticles in the broth can be estimated as the difference between the number of particles released from gel and the number of particles internalized by cells (Equation (10)). Both equations are controlled by a mass transfer coefficient (K_s_a_x_) where a depends on the geometry which is different for gel release (planar surface of a cylinder) and bacteria uptake (spherical)

(10) $$\frac{{\mathrm{dC}}_{\mathrm{broth}}}{\mathrm{dt}}=K_{l}a_{1}\cdot \left(C_{\mathrm{int}}-C_{\mathrm{broth}}\right)-K_{l}a_{2}\cdot \left(C_{\mathrm{broth}}-C_{s}\right)$$

The concentration in the interface (C_int_) can be estimated as the ratio among the mass of AgLeNPs release and a differential volume (discretization method). The concentration in the surface of bacteria (C_s_) will be considered as the dose (D) for bacteria uptake because it is the real available concentration for this phenomenon.

### 3.6. Parameter Estimation

Parameter estimation was carried out in gPROMS 5.0.2 (PSE), by using the survival experimental data. The variance model selected was constant and the sensor for parameter estimation was fixed as a function of the variable survival (S), in a range from 10^−5^ to 0.1. Two parameters (from release kinetics) were estimated: k and n. Boundary conditions were set for each parameter estimation process. Iterations for k determination were in the range from 0 to 0.9; and iterations for n determination were in the range from 0 to 1.

For statistical analysis, weighted residuals were calculated and compared with chi-square value (χ^2^) for a confidence interval of 95%. It is generally assumed that model effectively predicts the experimental data when weighted residuals are less than chi-square value. For cases where weighted residuals are greater than chi-square, the model may need some changes to better describe the experimental data [36].

## 4. Conclusions

Silver-coated nanoparticles with bactericidal effect were synthetized by using a green technique with a fructose polymer (levan). A loading capacity of 29% was obtained while producing spherical nanoparticles with a size of about 35 nm. These levan-silver capped nanoparticles showed a bactericidal effect with both Gram-negative or Gram-positive bacteria, although a less effectiveness was found for Gram-positive due to the peptidoglycan wall. Moreover, it was found that bacterial survival followed a single-hit/multiple-target model (dose-response behaviour) with a silver lethal dose of 12.6 ppm for *B. subtilis* and of 6.8 ppm for *E. coli*.

Polymer-silver nanoparticles were also introduced in an alginate gel to produce a further silver delivery system that can be useful for different applications. The gel was able to reduce the bacterial survival up to 20% in 5–10 h depending on the bacteria genera. Based on dispersion axial phenomenon, silver release and nanoparticles diffusion to combine nanoparticles silver release from inside the gel to its “killing” effect, a model was successfully developed to predict both silver release and lethal dose for different bacteria type. This general model was able to predict accurately silver release and the tendency of the bactericidal effect for Gram positive and Gram negative bacteria (adjustable parameters are required to get an exact bactericidal effect). However, this model provides a complete knowledge for the potential use of a silver-gel for wound dressing applications.

## Figures and Tables

**Table 1:** Parameters estimated and statistical measurements.

Strain	AgLeNPs Concentration (µg/mL)	k (s^−n^)	*p*	Weight Residuals	χ^2^	Number of NLP Iterations	Time for Estimation (s)
*E. coli*	50	0.135	0.266	7.99	11.07	57	17
	80	0.205	0.181	6.99	9.49	64	21
	110	0.328	0.116	3.11	11.10	36	10
*B. subtilis*	50	0.715	0.203	0.82	12.59	20	7
	80	0.900	0.129	0.51	11.07	27	8
	110	0.156	0.262	2.25	12.56	27	8

**Table 2:** Parameters used for simulations.

Parameter	Value	Unit	Reference/Source
dp	0.017	m	Measurement (microwell plate)
Dif	1.23 × 10^−11^	m^2^/s	Calculated from Stokes-Einstein equation
a_1_	3 × 10^6^	m^−1^	Calculated from gel geometry
a_2_	2.27 × 10^3^	m^−1^	Calculated from bacteria diameter
S_ag_	15.7	s^−1^	Set experimental (150 rpm)
ρ_liq_	1000	kg/m^3^	Tabulated value for water
ρ_gel_	1064	kg/m^3^	Determined experimentally
V_gel_	0.1	mL	Set experimental
V_well_	0.9	mL	Set experimental
µ	0.001	kg/m·s	Tabulated value for water
T	298	K	Set experimental
r_H_	36	nm	Determined experimentally (DLS)
k_b_	1.38 × 10^−23^	J/K	Constant
**Initial Conditions**			
C_broth_	0	µg/mL	
C_s_	0	µg/mL	
M_out_	0	µg	

## Supplementary Materials

Supplementary materials can be found at https://www.mdpi.com/1422-0067/20/6/1502/s1.

## Abbreviations

A	Area for NPs release (m^2^), π·r_w_^2^
a_1_	Specific area for mass transfer gel to liquid (m^2^/m^3^)
a_2_	Specific area for mass transfer liquid to bacteria surface (m^2^/m^3^)
C_broth_	Concentration of NPs in culture broth (mg/mL)
C_in_	Average concentration of NPs inside gel (mg/mL)
C_int_	Concentration of NPs in the interface liquid-gel (mg/mL)
C_NPS_	Concentration of NPS (mg/mL)
C_s_	Concentration of nanoparticles in the surface of bacteria (mg/mL)
D	Silver dose (ppm)
D_0_	Lethal dose (ppm)
D_ef_	Effective diffusion coefficient in gel (m^2^/s)
Dif	Diffusion coefficient (m^2^/s)
D_w_	Well diameter (m)
k	Kornsmeyer-Peppas constant 1 (s^−n^)
K_b_	Boltzmann constant (J/K)
K_l_	Mass transfer coefficient (m/s)
M_in_	Mass of nanoparticles in gel-solid medium (mg)
M_out_	Mass of nanoparticles in liquid medium (mg)
M_tot_	Total mass of nanoparticles in the well (mg)
n	Number of targets in bacteria
N_NPS_	Number of nanoparticles (mol)
p	Kornsmeyer-Peppas constant 2
Re	Reynolds number, η/(ρ_liq_· Dif)
r_H_	Hydrodynamic radious of nanoparticles (m)
r_w_	Well radious (m)
S	Survival percentage (%)
S_ag_	Agitation speed in orbital shaker (s^−1^)
Sc	Scmidtt number, ρ_liq_·S_ag_·d_w_^2^/η
Sh	Sherwood number, d_w_·k_l_/Dif
T	Temperature (K)
t	Time (s)
v_r_	Release velocity (mg/mL·s)
µ	Fluid viscosity (kg/m·s)
ρ_gel_	Gel density (kg/m^3^)
ρ_liq_	Liquid-broth density (kg/m^3^)
