# Supplementary material for: Levan-Capped Silver Nanoparticles for Bactericidal Formulations: Release and Activity Modelling
Source: Int J Mol Sci. 2019 Mar 26;20(6):1502. doi: 10.3390/ijms20061502 (PMC6471909; doi:10.3390/ijms20061502)
Supplement: Supplementary file 1 [file ijms-20-01502-s001.pdf]

Supplementary material

1. Model fitting DOSE-RESPONSE

Correspondece in manuscript: section 3.2.

Experimental data were fitted to three different models for dose-response curves. A linearization of the equation was performed when it was needed. *E.coli* data is represented in blue (and the equations derived) whereas *B. subtilis* data is represented in orange (and the equations derived)

1. Single-hit/single-target

$$S = e^{-\alpha D}$$

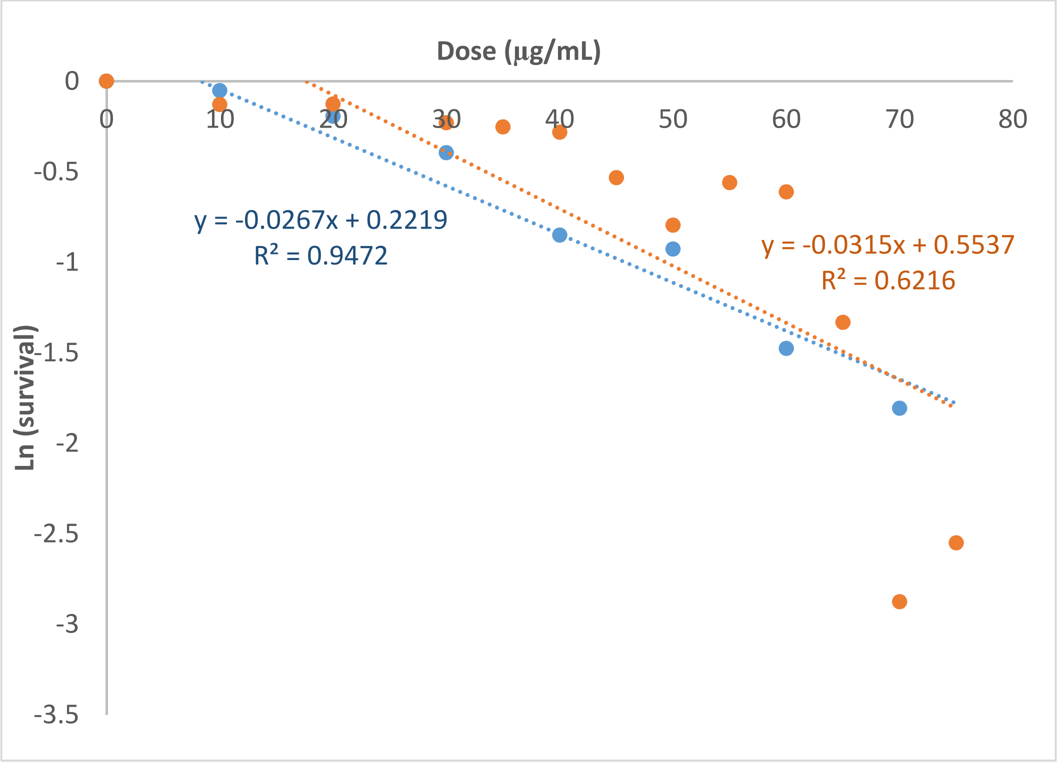

NO FIT

2. ***Multiple-hit/single-target***

$$S = e^{-(\alpha D + \beta D^2)}$$

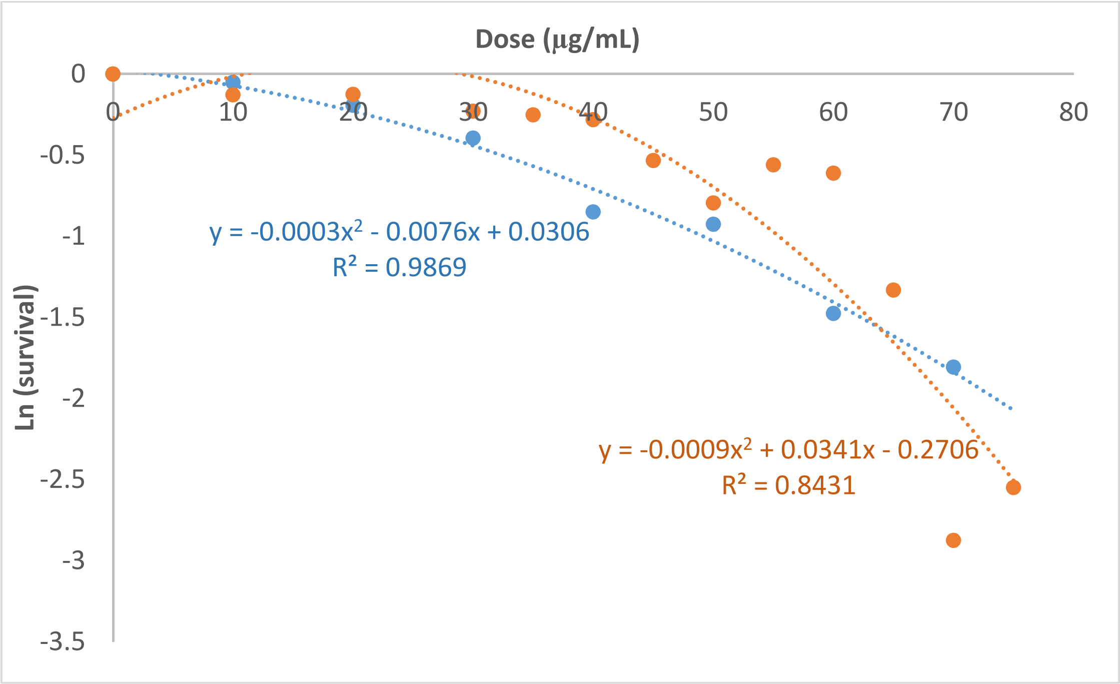

<u>NO FIT</u>

3. ***Single-hit/Multiple-target (SELECTED)***

$$S = 1 - (1 - e^{-\left(\frac{D}{Do}\right)})^n$$

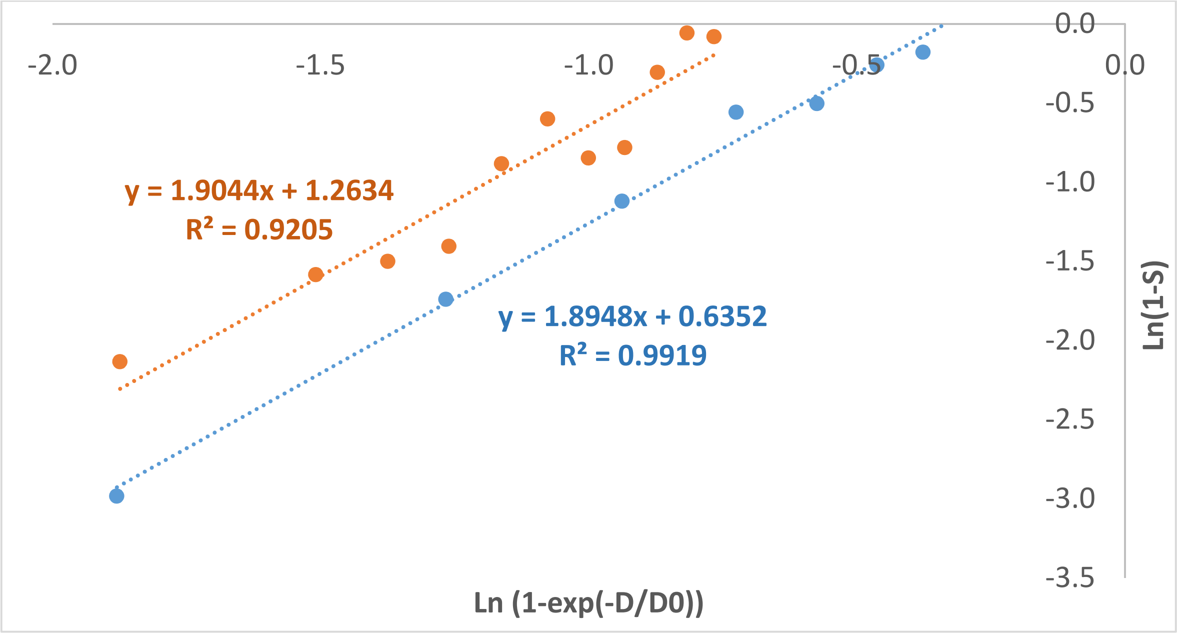

2. Statistical Test: Wilcoxon

a) Dose-response curves: Is there difference between Gram + and Gram - bacteria?

Dose (ppm)	E. coli	B. subtilis	Difference	Positive/ negative	Absolute value Difference	Rank	Singed Rank
**1**	0,9494	0,8795	-6,99E-02	-1	0,0699	3	-3
**2**	0,8245	0,8816	5,71E-02	1	0,0571	2	2
**3**	0,6738	0,7949	1,21E-01	1	0,1211	5	5
**4**	0,4274	0,7548	3,27E-01	1	0,3274	7	7
**5**	0,3959	0,4514	5,55E-02	1	0,0555	1	1
**6**	0,2286	0,5425	3,14E-01	1	0,3139	6	6
**7**	0,1642	0,2639	9,97E-02	1	0,0997	4	4
							25
							-3

For seven trials: critical value: 3 (significance level 0,05)

At least one sum: -3, in absolute value → 3 ≤ 3 → Significant difference

b) Survival curves: Is there difference between concentrations on survival

One case as an example: *Differences in E. Coli for 50 and 80 ppm AgNPs*

Time (h)	50 ppm	80 ppm	Difference	Positive/ Negative	Absolute value difference	Rank	Singed Rank	
1	1	1	0,00E+00	-1	0	1	-1	
2	0,342	0,554	2,12E-01	1	0,212	7	7	
3	0,526	0,533	7,00E-03	1	0,007	4	4	
4	0,456	0,458	2,00E-03	1	0,002	2	2	
5	0,404	0,4	-4,00E-03	-1	0,004	3	-3	
6	0,199	0,361	1,62E-01	1	0,162	6	6	
7	0,358	0,422	6,40E-02	1	0,064	5	5	
							24	Positive sum
							-4	Negative sum

For seven trials: critical value: 3 (significance level 0,05)

Both sums, in absolute value: 4 and 24. 4 > 3, 24 > 3 → There is no significant difference
